# Supplementary material for: Igf2bp3 maintains maternal RNA stability and ensures early embryo development in zebrafish
Source: Commun Biol. 2020 Mar 3;3:94. doi: 10.1038/s42003-020-0827-2 (PMC7054421; doi:10.1038/s42003-020-0827-2)
Supplement: Supplementary file 1 — Supplementary Information [file 42003_2020_827_MOESM1_ESM.pdf]

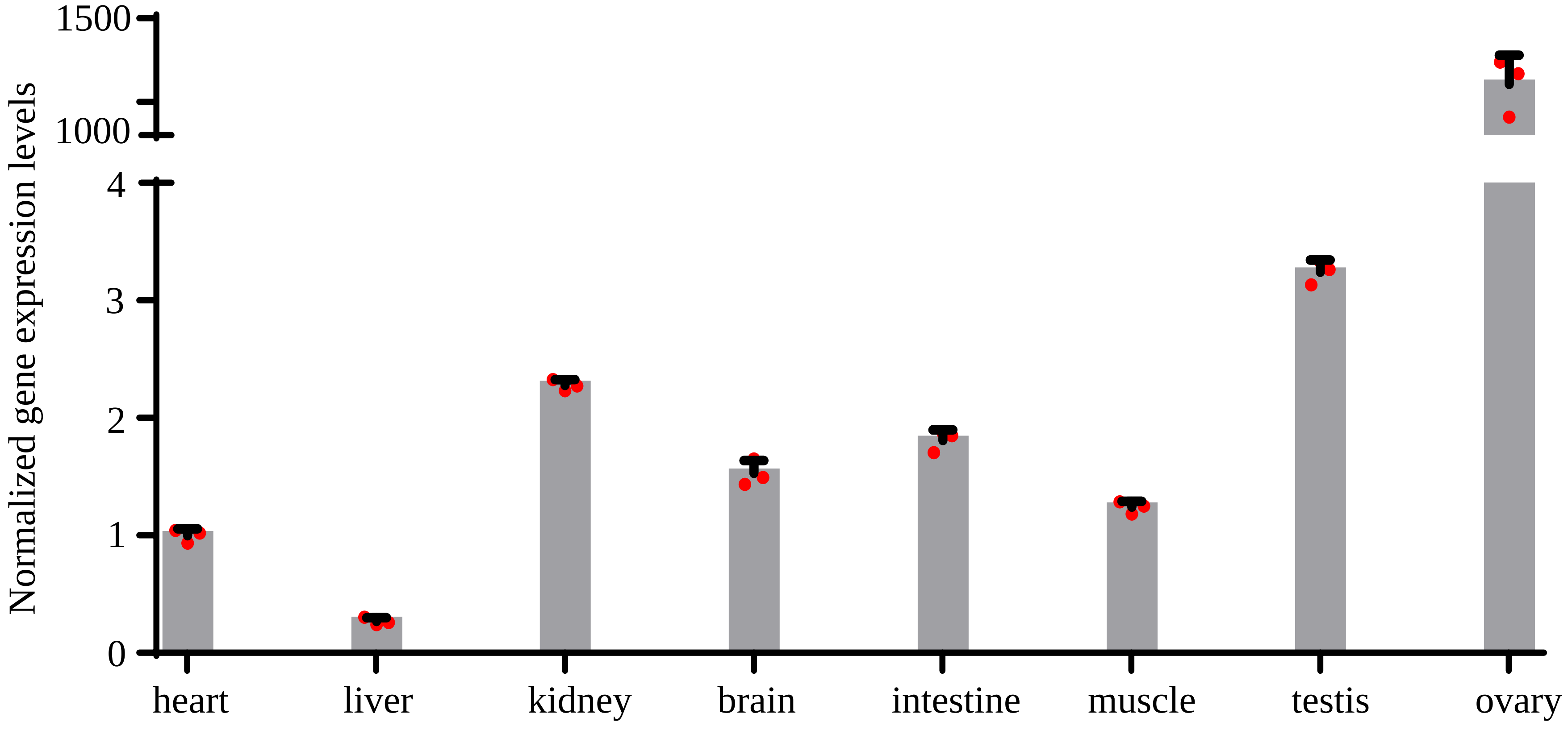

Supplementary Figure 1: *igf2bp3* mRNA expression in zebrafish tissues. qRT-PCR was performed to detect *igf2bp3* mRNA in several tissues including heart, liver, kidney, brain, intestine, muscle, testis and ovary.

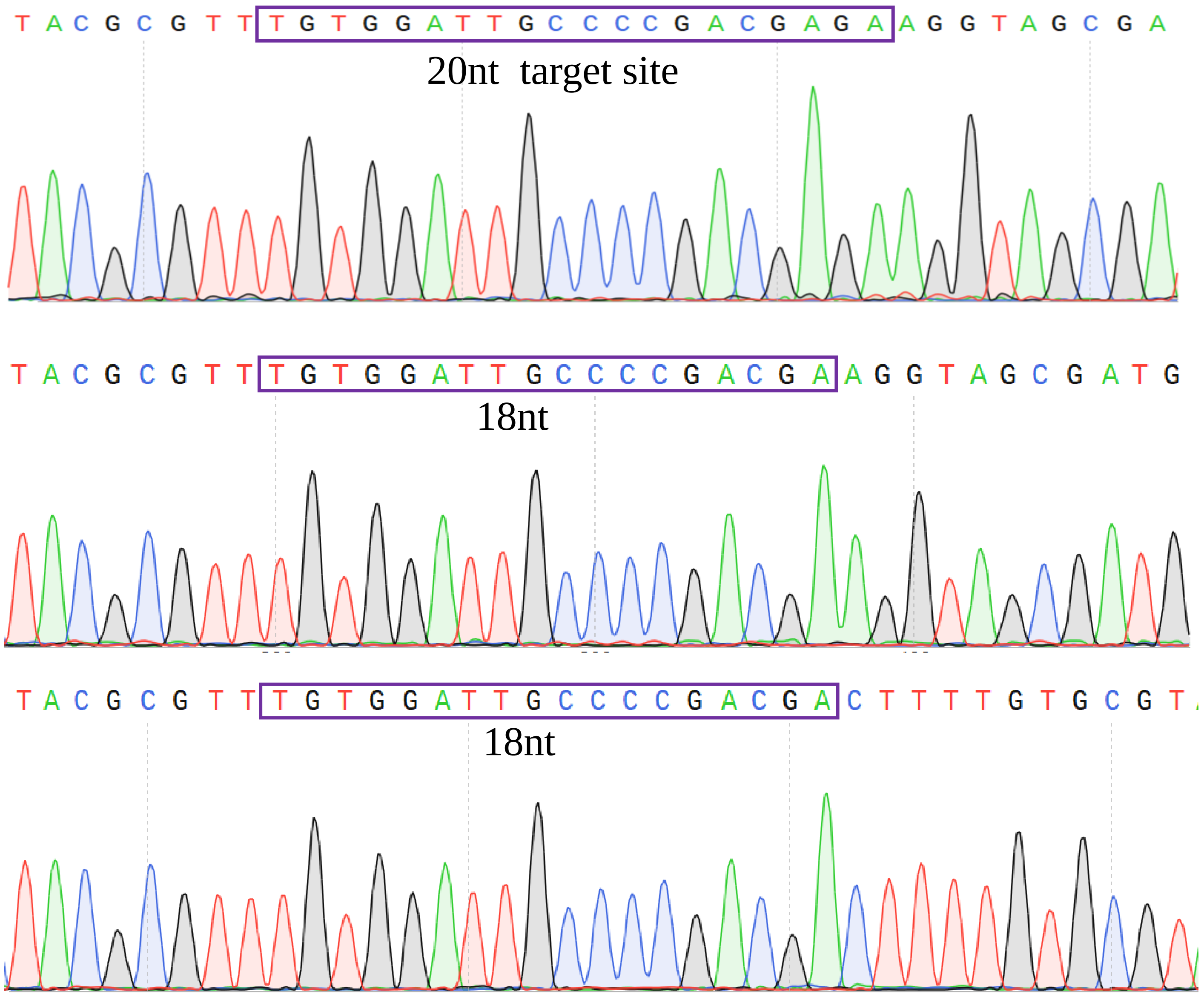

Wild-type

M*igf2bp3* (2nt deletion)

M*igf2bp3* (41nt deletion)

Supplementary Figure 2: The Sanger sequencing of the PCR products near the CRISPR/Cas9 target site in the F2 generation embryos.

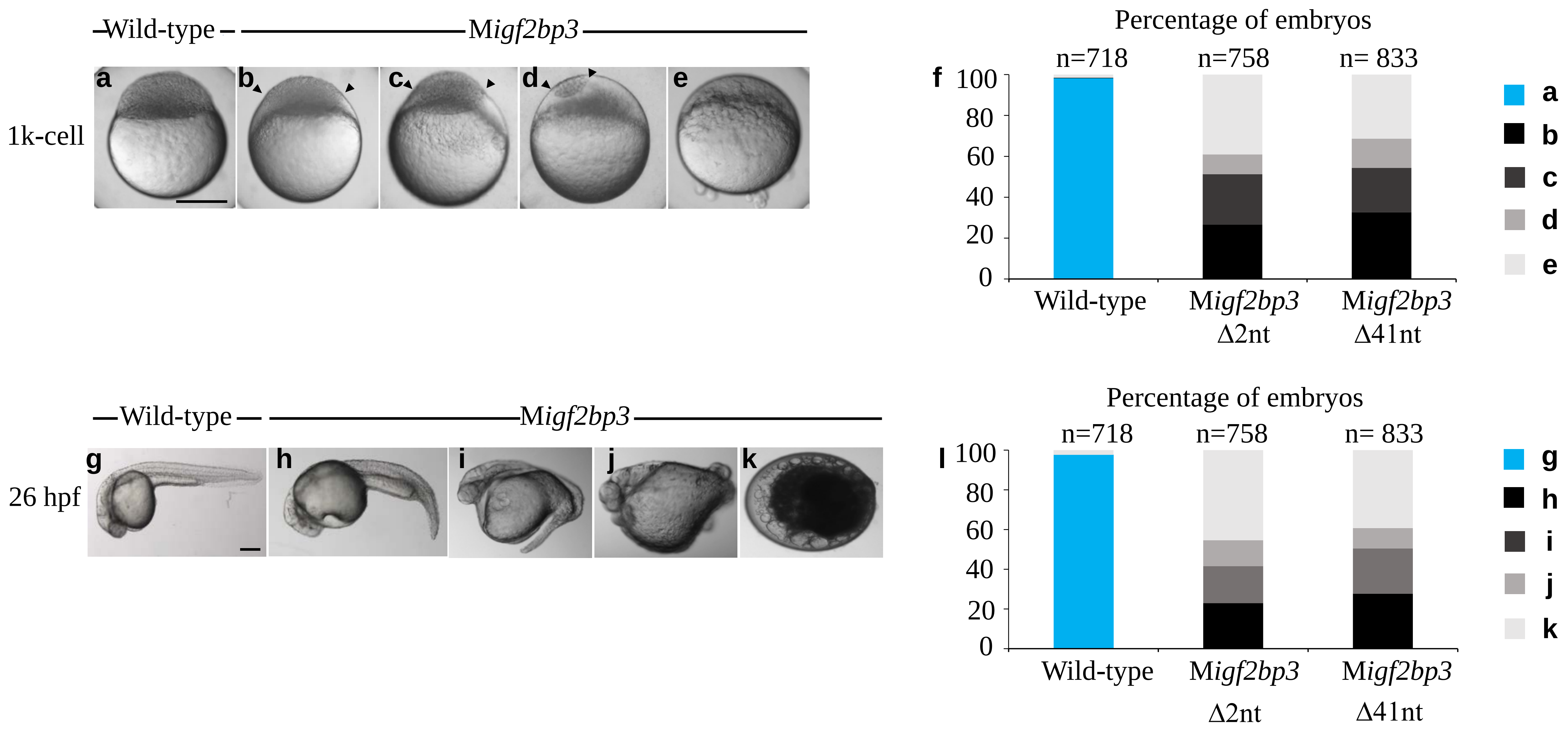

Supplementary Figure 3: **M*igf2bp3* embryos display severe defects at 1k-cell and 26 hpf.** (**a-e**) The defective phenotypes of M*igf2bp3* embryos at 1k-cell stages. (**f**) Percentages of embryos according to the phenotypes classified in (**a-e**) in wild-type and M*igf2bp3* embryos. (**g-k**) The defective phenotypes of M*igf2bp3* embryos at 26 hpf. M*igf2bp3* embryos displayed different extents of deficiency, such as yolk sac defect (**h**), reduced anterior-posterior axis (**i-j**) and dead (**k**). (**l**) Percentages of embryos according to the phenotypes classified in (**g-k**) in wild-type and M*igf2bp3* embryos. Scale bars: 200 µm.

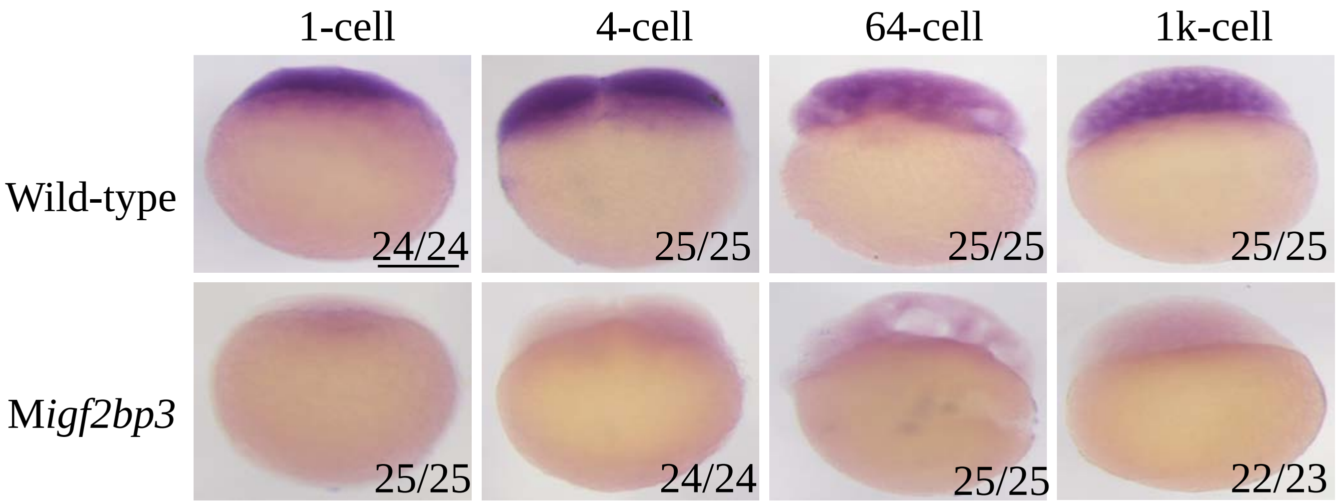

Supplementary Figure 4: **The expression pattern of *igf2bp3* mRNA in wild-type and M*igf2bp3* embryos**. *igf2bp3* mRNA was located at the animal pole during early zebrafish embryo development. The expression of *igf2bp3* was dramatically reduced in M*igf2bp3* embryos. Scale bars: 200 µm.

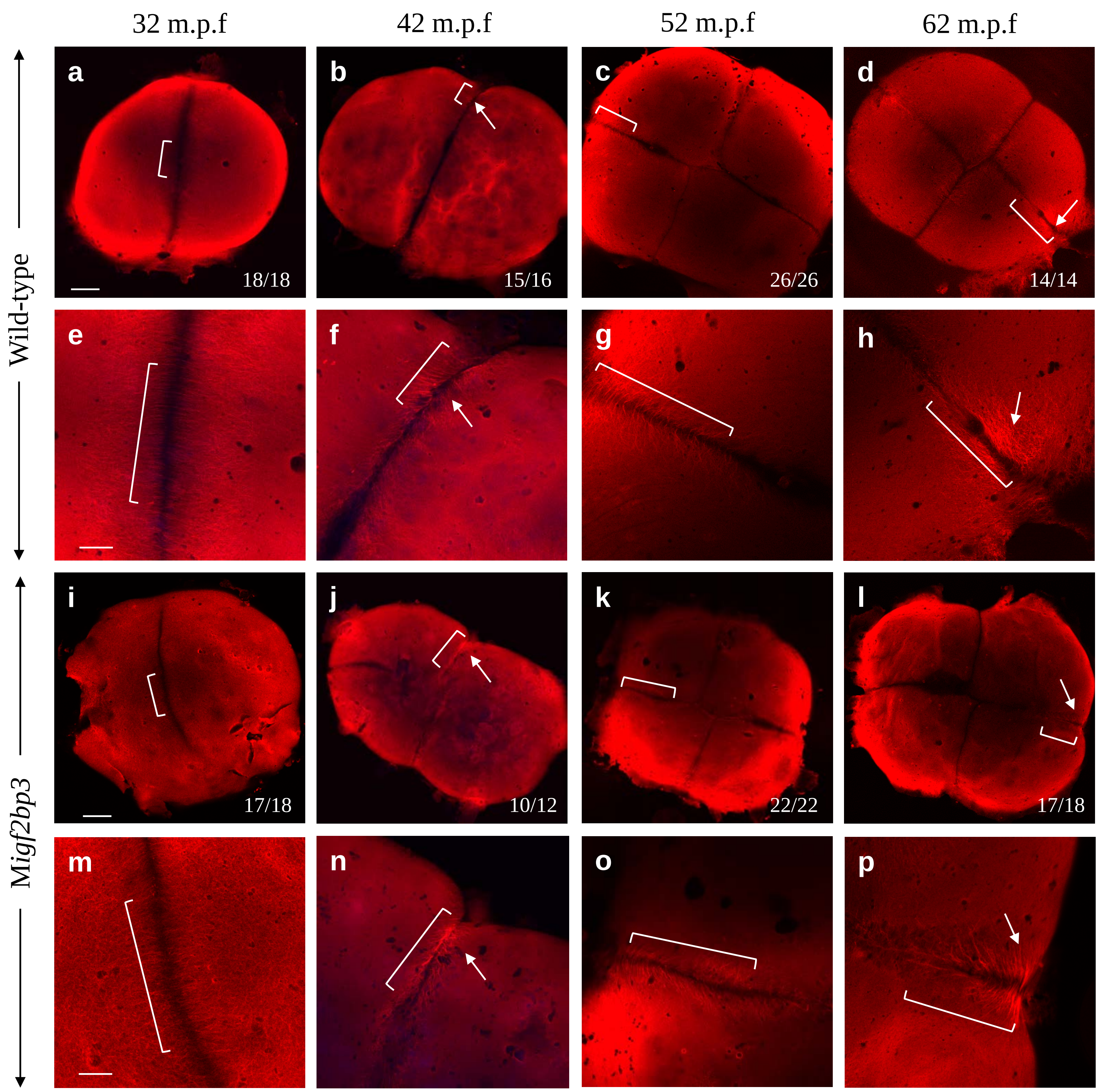

Supplementary Figure 5: **The furrow microtubule dynamics is unaffected in M*igf2bp3* embryos during early zebrafish embryogenesis.** Wild-type and M*igf2bp3* embryos were fixed at the indicated time points and labeled with an anti-α-tubulin antibody. **e-h** and **m-p** were high magnification images of **a-d** and **i-l** at the bracketed region. The bracketed region showed the tubules of the furrow microtubule array (FMA). As the furrow matures, FMA tubules progressively accumulate and become bulk enrichment in the distal region of embryos (arrows). Scale bars: 100 μm in **a-d, i-l** and 50 μm in **e-h**, **m-p**.

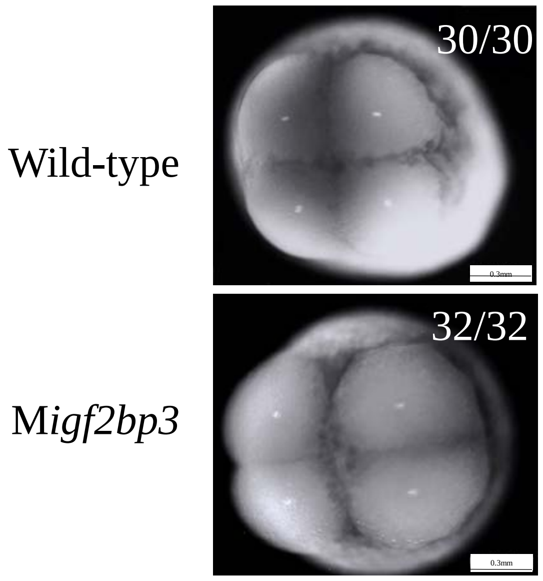

Supplementary Figure 6: **M*igf2bp3* embryos display regular nuclear division.** The embryos displayed regular nuclear morphology between the wild-type and M*igf2bp3* embryos. Scale bars: 0.3 mm.

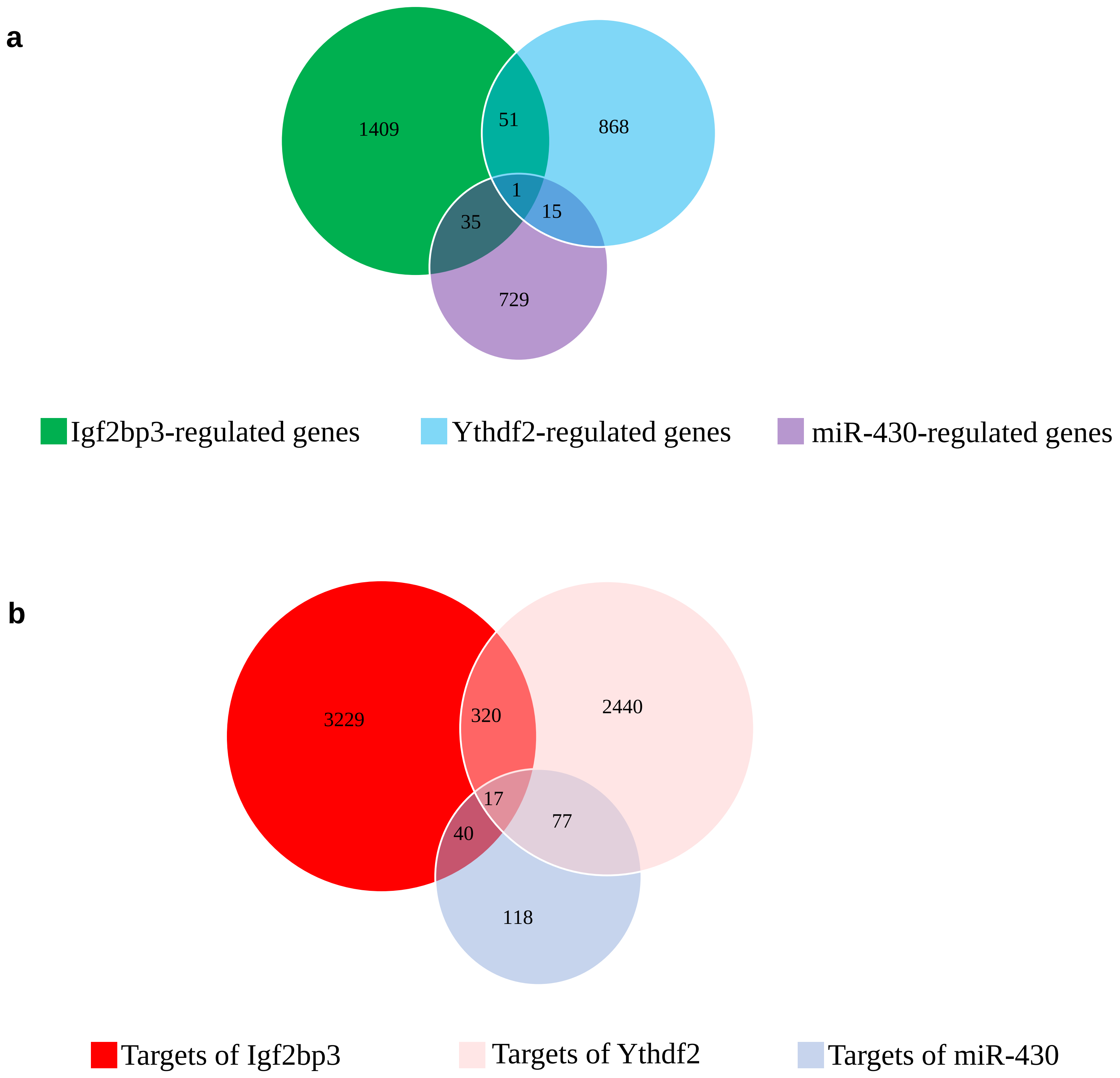

Supplementary Figure 7: Venn diagram showing the overlap of regulated genes (a) and targets (b) of Igf2bp3, Ythdf2 and miR-430. Analyzed the mRNA degradation profile of M*igf2bp3* embryos and miR-430- or Ythdf2-mediated degradation profile, and obtained the regulatory genes of Igf2bp3, Ythdf2 and miR-430.

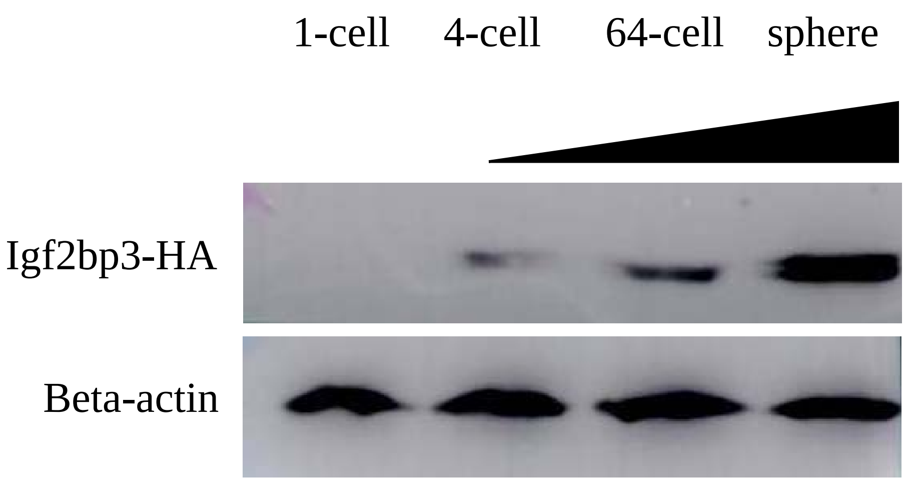

Supplementary Figure 8: Detecting the Igf2bp3-HA protein expression in embryos. *igf2bp3-HA* mRNA was injected into the fertilized eggs of wild-type zebrafish and deteced its protein expression at different embryonic stages. Beta-actin was used as the control.

Supplementary Table 1 Information of the primers used for probes and qRT-PCR		
Primers	Sequences(5′–3′)	Size of the products (bp)
**The primers used for probes**		
igf2bp3-F	AGTGGGAAGTTTTAGACGGGTTG	1227
igf2bp3-R	TAATACGACTCACTATAGGGCAGCGAATGATGGCACTTTTAT	1227
**The primers used for qRT-PCR**		
dazl-F	GCATCGTCAGGGTTTTCCG	122
dazl-R	TCTCGTTCTCATCCACCTTCAT	122
dnd1-F	TGACCCTCTTACAGCCTCGG	228
dnd1-R	GCCACAACCTCTTTCCCTTT	228
igf2bp3-F	AGCGAGTGGAGGGATTTCA	192
igf2bp3-R	ATTGACGCACCAGCGAAGC	192
buc-F	CCACAAGTGACCCAAGAGCG	135
buc-R	CCTACCACCACCAACATAAACA	135
aurkb-F	AAGAGGGAGTGGAGCATCAG	90
aurkb-R	GGAAGTAGTTGTAGAAGCGAAGG	90
srsf6a-F	CCTGTTCGCACTGAGTATCG	248
srsf6a-R	CGTCGTCTTCGTGGCTTAT	248
bbip1-F	TGTTCCCACTATGGTCCT	125
bbip1-R	CCTTCAAAGCCAGTTCCT	125
tmem230b-F	AACAGTGGAGTCAGATAC	162
tmem230b-R	TAACAGGAGAGACCCGAT	162
18s-F	AGGGACAAGTGGCGTTCAG	156
18s-R	AATCACGAGCGGGGTTCA	156
actb1-F	GATGGGAACCGCTGCCTC	91
actb1-R	ACGGAAACGCTCATTGCC	91

Supplementary Table 1 The information of the primers used for probes and qRT-PCR.
