# Supplementary material for: Igf2bp3 maintains maternal RNA stability and ensures early embryo development in zebrafish
Source: Commun Biol. 2020 Mar 3;3:94. doi: 10.1038/s42003-020-0827-2 (PMC7054421; doi:10.1038/s42003-020-0827-2)
Supplement: Supplementary file 2 — Description of Additional Supplementary Files [file 42003_2020_827_MOESM2_ESM.pdf]

Description of Additional Supplementary Files

Supplementary Data 1. The data of RNA-seq (Wild-type and *igf2bp3* mutant embryos) and Igf2bp3-HA RIP-seq .

Supplementary Data 2. The source data used for the following Figures:

Fig.1**d**

Fig. 5**b**

Fig. 6**b**,6**c**

Supplementary Figure 1

Supplementary Figure 3**f**, 3**l**

Supplementary Figure 8
