# Supplementary material for: Igf2bp3 maintains maternal RNA stability and ensures early embryo development in zebrafish
Source: Commun Biol. 2020 Mar 3;3:94. doi: 10.1038/s42003-020-0827-2 (PMC7054421; doi:10.1038/s42003-020-0827-2)
Supplement: Supplementary file 5 — Reporting Summary [file 42003_2020_827_MOESM5_ESM.pdf]

nature research

Corresponding author(s): Jie Mei

Last updated by author(s): Jan 29, 2020

Reporting Summary

Nature Research wishes to improve the reproducibility of the work that we publish. This form provides structure for consistency and transparency in reporting. For further information on Nature Research policies, see Authors & Referees and the Editorial Policy Checklist.

Statistics

For all statistical analyses, confirm that the following items are present in the figure legend, table legend, main text, or Methods section.

n/a	Confirmed	
☐	☒	The exact sample size (*n*) for each experimental group/condition, given as a discrete number and unit of measurement
☐	☒	A statement on whether measurements were taken from distinct samples or whether the same sample was measured repeatedly
☐	☒	The statistical test(s) used AND whether they are one- or two-sided<br>*Only common tests should be described solely by name; describe more complex techniques in the Methods section.*
☒	☐	A description of all covariates tested
☒	☐	A description of any assumptions or corrections, such as tests of normality and adjustment for multiple comparisons
☒	☐	A full description of the statistical parameters including central tendency (e.g. means) or other basic estimates (e.g. regression coefficient) AND variation (e.g. standard deviation) or associated estimates of uncertainty (e.g. confidence intervals)
☒	☐	For null hypothesis testing, the test statistic (e.g. *F*, *t*, *r*) with confidence intervals, effect sizes, degrees of freedom and *P* value noted<br>*Give P values as exact values whenever suitable.*
☒	☐	For Bayesian analysis, information on the choice of priors and Markov chain Monte Carlo settings
☒	☐	For hierarchical and complex designs, identification of the appropriate level for tests and full reporting of outcomes
☒	☐	Estimates of effect sizes (e.g. Cohen's *d*, Pearson's *r*), indicating how they were calculated

Our web collection on statistics for biologists contains articles on many of the points above.

Software and code

Policy information about availability of computer code

Data collection	All Illumina sequencing data in this study were collected by the manufacturer's software following the manufacturer's pipeline.
Data analysis	R statistical language(version 3.6) and GraphPad Prism 7 were used for data analysis.

For manuscripts utilizing custom algorithms or software that are central to the research but not yet described in published literature, software must be made available to editors/reviewers. We strongly encourage code deposition in a community repository (e.g. GitHub). See the Nature Research guidelines for submitting code & software for further information.

Data

Policy information about availability of data

All manuscripts must include a data availability statement. This statement should provide the following information, where applicable:

- Accession codes, unique identifiers, or web links for publicly available datasets
- A list of figures that have associated raw data
- A description of any restrictions on data availability

Raw sequence data generated during this study can be accessed at NCBI SRA database under Bioproject accession number PRJNA565584.(www.ncbi.nlm.nih.gov/bioproject/PRJNA565584). All data are available from the corresponding author at request on reasonable request.

Field-specific reporting

Please select the one below that is the best fit for your research. If you are not sure, read the appropriate sections before making your selection.

☒ Life sciences ☐ Behavioural & social sciences ☐ Ecological, evolutionary & environmental sciences

Life sciences study design

All studies must disclose on these points even when the disclosure is negative.

Sample size	Sample size was indicated in the figure legends and source data of each experiment. For gene expression, about 30 embryos of wild-type and mutant at each stage were used. 5 wild-type adult zebrafish were used for gene expression in tissues. 5 adult zebrafish in wild-type and mutant were used for sections and H&E staining of ovary. About 30 eggs and embryos were collected in each group to prepare for RNA-seq. No less than 1000 zebrafish embryos were prepared for RIP.
Data exclusions	No data were excluded from the analyses.
Replication	All attempts at replication were successful.
Randomization	Sample allocation into groups was random.
Blinding	There is no blinding in the study, because the phenotypes are distinct between the mutant and control embryos.

Reporting for specific materials, systems and methods

We require information from authors about some types of materials, experimental systems and methods used in many studies. Here, indicate whether each material, system or method listed is relevant to your study. If you are not sure if a list item applies to your research, read the appropriate section before selecting a response.

Materials & experimental systems

n/a	Involved in the study	
☐	☒	Antibodies
☒	☐	Eukaryotic cell lines
☒	☐	Palaeontology
☐	☒	Animals and other organisms
☒	☐	Human research participants
☒	☐	Clinical data

Methods

n/a	Involved in the study	
☒	☐	ChIP-seq
☒	☐	Flow cytometry
☒	☐	MRI-based neuroimaging

Antibodies

Antibodies used	All antibodies used are commercially available.<br>rabbit Monoclonal to HA-tag, catalogue #3724, lot #8, Cell Signaling Technology.<br>rabbit polyclonal to β-Catenin, catalogue # ab16051, Abcam.<br>IgG control, catalogue # NI01, lot #3010039, Merck Millipore.<br>anti-Rabbit IgG Alexa Fluor® Plus 488-conjugated secondary antibody, catalogue # A32731, lot #SE250296, Invitrogen.<br>anti-β-actin antibody, AC026,ABclonal.
Validation	All experimental antibodies in this study are commercially available and are validated for the species and technique, and the validation data is accessible from vendor's website.

Animals and other organisms

Policy information about studies involving animals; ARRIVE guidelines recommended for reporting animal research

Laboratory animals	Zebrafish embryos were all used between 0 and 26 hours post fertilization and the adult zebrafish were about 90 day post fertilization.
Wild animals	N/A
Field-collected samples	N/A
Ethics oversight	All experiments involving zebrafish were approved and in compliance with the requirements of the IACUC of Huazhong Agricultural University (HZAUFI-2015-006).

Note that full information on the approval of the study protocol must also be provided in the manuscript.
